# Revealing the Dynamic Modulations That Underpin a Resilient Neural Network for Semantic Cognition: An fMRI Investigation in Patients With Anterior Temporal Lobe Resection

**DOI:** 10.1093/cercor/bhy116

**Published:** 2018-06-06

**Authors:** Grace E Rice, Helen Caswell, Perry Moore, Matthew A Lambon Ralph, Paul Hoffman

**Affiliations:** 1 Neuroscience and Aphasia Research Unit (NARU), University of Manchester, Manchester, UK; 2 Department of Clinical Neuropsychology, Salford Royal Hospital, Manchester, UK; 3 Department of Clinical Neuropsychology, The Walton Centre NHS Foundation Trust, Liverpool, UK; 4 Centre for Cognitive Ageing and Cognitive Epidemiology (CCACE), Department of Psychology, University of Edinburgh, Edinburgh, UK

**Keywords:** anterior temporal lobectomy, conceptual knowledge, laterality, semantic memory, temporal lobe epilepsy

## Abstract

One critical feature of any well-engineered system is its resilience to perturbation and minor damage. The purpose of the current study was to investigate how resilience is achieved in higher cognitive systems, which we explored through the domain of semantic cognition. Convergent evidence implicates the bilateral anterior temporal lobes (ATLs) as a conceptual knowledge hub. While bilateral damage to this region produces profound semantic impairment, unilateral atrophy/resection or transient perturbation has a limited effect. Two neural mechanisms might underpin this resilience to unilateral ATL damage: 1) the undamaged ATL upregulates its activation in order to compensate; and/or 2) prefrontal regions involved in control of semantic retrieval upregulate to compensate for the impoverished semantic representations that follow from ATL damage. To test these possibilities, 34 postsurgical temporal lobe epilepsy patients and 20 age-matched controls were scanned whilst completing semantic tasks. Pictorial tasks, which produced bilateral frontal and temporal activation, showed few activation differences between patients and control participants. Written word tasks, however, produced a left-lateralized activation pattern and greater differences between the groups. Patients with right ATL resection increased activation in left inferior frontal gyrus (IFG). Patients with left ATL resection upregulated both the right ATL and right IFG. Consistent with recent computational models, these results indicate that 1) written word semantic processing in patients with ATL resection is supported by upregulation of semantic knowledge and control regions, principally in the undamaged hemisphere, and 2) pictorial semantic processing is less affected, presumably because it draws on a more bilateral network.

## Introduction

One critical feature of any well-engineered system is its resilience to perturbation and minor damage. How resilience is achieved in higher cognitive systems is important both for cognitive and clinical neuroscience. Although rarely considered in laboratory-based explorations of higher cognition, in everyday life we are faced with and are resilient to variations in task difficulty, degraded stimuli, etc. Likewise after partial brain damage or perturbation, patients can sometimes show impressive resilience and recovery. The current study examined the dynamic changes that support resilience in the domain of semantic cognition—both in patients with partial damage to the semantic representational system and in the healthy system following variation in task difficulty.

Unilateral resection is the primary surgical treatment for drug-resistant temporal lobe epilepsy (TLE) (Wiebe et al. 2001). Surgical resection alleviates seizure activity in 80–90% of patients making it the gold standard of treatment (Wiebe et al. 2001). Behaviorally, patients with unilateral anterior temporal lobe (ATL) resection exhibit short-term memory problems and relatively mild semantic memory impairments (Wilkins and Moscovitch 1978; Alpherts et al. 2006; Lambon Ralph et al. 2012; Willment and Golby 2013; Sidhu et al. 2015). This mild semantic impairment is shown through slower response times on semantic tasks (e.g., picture naming, word-picture matching, synonym judgment), rather than marked decreases in accuracy (Wilkins and Moscovitch 1978; Lambon Ralph et al. 2012; Rice et al. 2018), although on more challenging/specific level semantic concepts decreases in accuracy performance are found. This mild semantic impairment in patients with unilateral ATL resection is in contrast to the severe semantic impairment shown after bilateral ATL damage/resection (Terzian and Ore 1955; Lambon Ralph et al. 2010, 2012; Mion et al. 2010; Zhao et al. 2017). This pattern of results was also shown in seminal nonhuman primate work (Brown and Schafer 1888; Kluver and Bucy 1937, 1938), in which unilateral resection resulted in transient deficits, whereas bilateral resection resulted in severe multimodal semantic and episodic memory impairments. Converging evidence from functional neuroimaging and neurostimulation studies indicates that the bilateral ATLs play a critical and central role in semantic representation (Pobric et al. 2010; Visser, Embleton et al. 2010; Shimotake et al. 2014; Rice, Lambon Ralph, and Hoffman 2015; Lambon Ralph et al. 2017). The discrepancy in behavioral performance in patients with unilateral versus bilateral ATL damage/atrophy imply that this system is configured to be robust and relatively resistant to unilateral damage (Schapiro et al. 2013). Despite this, the neuronal mechanisms underlying this resilience are not clear. Two nonexclusive and potentially related explanations for the robustness of the semantic system are: 1) after unilateral resection the remaining contralateral ATL changes its activation in order to compensate; and/or 2) performance on semantic tasks is maintained through changes in the activation in other regions of the semantic network.

The first potential explanation for the robustness of the semantic system is that the system has a certain amount of redundancy when semantic representations are distributed across both left and right ATLs. As such it would follow that, in patients with unilateral damage/resection, the undamaged contralateral ATL would still retain considerable representational effectiveness. As a consequence, the semantic impairment in TLE is primarily shown through slower response times, rather than as marked decreases in accuracy (Lambon Ralph et al. 2010; Schapiro et al. 2013). Formal computational explorations of a bilaterally configured ATL system not only showed the same behavioral difference between unilateral (limited deficits) versus bilateral damage (considerable impairment) even when total damage is matched, but also found a second important factor (Schapiro et al. 2013). Specifically, damage to a local neural network not only weakened the distributed representation but it also resulted in activation noise which can be propagated to connected units. Accordingly, in unilateral lesions, noise was propagated to other ipsilateral hub units but less was transmitted to the distant contralateral hub. After bilateral damage, noise was pervasive throughout the representational system.

Evidence for a critical role of the contralateral hemisphere after unilateral perturbation has been observed in concurrent fMRI-TMS studies in healthy participants (Binney and Lambon Ralph 2015; Jung and Lambon Ralph 2016). Repetitive transcranial magnetic stimulation to the left ATL diminished activation in this region during semantic processing (mimicking unilateral ATL damage) and increased activation in the nonstimulated right ATL. Jung and Lambon Ralph (2016) also employed effective connectivity analysis and found that it is not just the increased activation of the contralateral ATL that is important in maintaining normal semantic performance, but the connectivity between the left and right ATLs. Specifically, after left ATL theta-burst stimulation, in addition to the activation changes in the left and right ATLs, there was increased effective connectivity from the right ATL to the left ATL (Jung and Lambon Ralph 2016). In a combined fMRI-neuropsychological study, Warren et al. (2009) also highlighted the importance of the connectivity between the ATLs in supporting language performance. In a group of chronic poststroke aphasic patients, these authors observed an overall decrease in inter-ATL connectivity. However, patients who exhibited greater preservation of this connectivity showed better language/comprehension performance.

A second type of explanation for the robustness of semantic performance is that there is increased activation of the regions involved in control and selection of semantic knowledge, thereby compensating for the reduced quality of semantic representations that follow from ATL dysfunction/damage. The inferior frontal gyrus (IFG) shows strong responses to increased semantic processing demand and has been implicated in the executive-control component of semantic cognition (Thompson-Schill et al. 1997; Badre and Wagner 2002; Noonan et al. 2013). In the context of patients with TLE, regions in the bilateral IFG were found to be upregulated in semantic tasks both before surgery (Billingsley et al. 2001; Koylu et al. 2006; Bonelli et al. 2012; Rosazza et al. 2013) and after ATL resection surgery (Backes et al. 2005; Noppeney et al. 2005; Koylu et al. 2006; Wong et al. 2009; Bonelli et al. 2012; Tivarus et al. 2012). This increase of IFG activation is not uniform; rather, activation in the IFG interacts with the site of resection. Patients who have undergone right ATL resection activate similar left IFG regions to control participants, but do so more strongly. In contrast, patients who have undergone left ATL resection show reduced activation in the left IFG during expressive semantic tasks and increased activation in the right IFG compared with controls (Backes et al. 2005; Koylu et al. 2006; Wong et al. 2009; Bonelli et al. 2012). This shift in IFG activation following left ATL resection has been shown to be behaviorally relevant, such that stronger activation in the right IFG is correlated with better naming performance after surgery (Bonelli et al. 2012).

In addition, upregulation of the IFG shows dynamic changes depending on the modality of the task. Episodic encoding of written words in preoperative left TLE patients has been associated with bilateral IFG activation, in comparison to the left-lateralized pattern of response in preoperative right TLE patients and control participants (Maccotta et al. 2007). Contrastively, encoding of nonverbal material generated right lateralized frontal responses in both left and right TLE patients and control participants (Maccotta et al. 2007). This study also showed that after surgery, the left TLE patients regained more of a left-lateralized activation pattern in the verbal encoding task (however, this neural change did not have a corresponding behavioral change in terms of accuracy on the task). This implies that changes in prefrontal activation after unilateral resection may interact with the task being performed, as well as the laterality of resection.

Within the IFG, there are regions hypothesized to underlie the cognitive control of language specifically versus others involved in domain-general cognitive control (Badre et al. 2005; Badre and D’Esposito 2009; Duncan 2010). This raises the possibility that recovery after unilateral resection may not be underpinned by semantic/language specific processes but by the upregulation of domain-general cognitive resources. Evidence for this in the context of stroke aphasia comes from Brownsett et al. (2014), who showed that during an auditory comprehension task stroke aphasic patients upregulated regions in the midline frontal cortex (encompassing the dorsal anterior cingulate cortex and superior frontal gyrus). This region forms part of the domain-general cognitive control network and was neuroanatomically distinct from the language-specific IFG activation in control participants when performing the same task. An extension of this line of thinking suggests that the domain-general cognitive control regions that are upregulated after damage are also the same regions healthy participants use during more challenging processing conditions (Sharp et al. 2004; Brownsett et al. 2014; Geranmayeh et al. 2014). In other words, changes in the system after damage are not random but may reflect intrinsic mechanisms present in the healthy brain. To test this hypothesis, Brownsett et al. (2014) tested control participants on a more challenging auditory comprehension task using noise-vocoded speech. Under these more demanding conditions, controls performed less accurately and, critically, showed increased activation in the same midline frontal regions that were upregulated in the stroke patients. Brownsett et al. (2014) concluded that recovery of language processing after stroke was driven by domain-general cognitive control regions, which are also recruited by healthy individuals in the context of increased processing demands. It is possible that similar mechanisms are engaged by resected TLE patients to compensate for the impaired function of the ATL semantic system.

This study was designed to investigate whether one or both of these resilience mechanisms (shift in the division of semantic labor across the ATLs; and/or engagement of executive control) is found in patients with resection for TLE. The specific goals and features of the study were as follows: 1) determine whether the increased activity in prefrontal regions reflects subregions associated with semantic executive processes or domain-general cognitive control. 2) Explore the relative engagement of ipsilateral and contralateral ATL/temporal regions We note that, in the small handful of previous fMRI explorations of patients with resection for TLE, the focus has primarily been on speech production (naming, verbal fluency, etc.) and on the changes observed in frontal regions. There is a paucity of data on the primary region of interest for TLE, namely, the ATL, though explorations using combined rTMS-fMRI suggest there might be important changes in this region (Schapiro et al. 2013; Binney and Lambon Ralph 2015; Jung and Lambon Ralph 2016). This lack of data might be related to a set of methodological challenges associated with successful fMRI investigations of the ATL including fMRI signal drop-out and distortion, the use of active baselines and ensuring a full field-of-view (Devlin et al. 2000; Visser, Jefferies, and Lambon Ralph 2010). Accordingly, all of these methodological issues were addressed in the current investigation, including the use of dual-echo EPI imaging to improve signal in the ATLs (Halai et al. 2014). 3) As noted above, previous explorations of episodic memory indicate that the fMRI changes are dynamic—changing according to modality of presentation. This has never been explored in the context of semantic function. Given the recent computational explorations of bilateral versus unilateral ATL-hub function, we predicted that a bilaterally distributed task (i.e., pictorial semantic processing) may be more robust to unilateral resection, compared with a relatively left-lateralized task (e.g., written word semantic processing).

These study goals were met by exploring whether changes in activation interacted with 1) side of resection (left vs. right ATL resection) and 2) the modality of the task (written words vs. pictures). Semantically related activation in the contralateral ATL and other regions in the semantic network were compared between resected TLE patients and a group of age-matched control participants. To explore whether control participants also upregulated a similar network of regions under increased task demands, a difficulty manipulation was employed in the control group. One important point to note is that the current study involved only postsurgical data from resected TLE patients. This means that there are a number of potential explanations for any difference found between patients and healthy controls, including the resection surgery itself and to the presence of long-standing epilepsy affecting functional networks prior to surgery. We consider these possibilities in more detail in the Discussion.

## Method

### Patients

A total of 34 patients who had a single “en-bloc” unilateral resection for medically intractable epilepsy took part in the study (17 left TLE, 17 right TLE). All patients underwent extensive neuropsychological testing (Table 1). Briefly, both the left and right TLE patients were matched on age (*t*[32] = 0.56, *P* = 0.58), education (*t*[32] = 0.80, *P* = 0.43), epilepsy duration (*t*[32] = 0.28, *P* = 0.78), age at surgery (*t*[32] = 0.27, *P* = 0.79), age at diagnosis (*t*[32] = 0.02, *P* = 0.98), and number of antiepileptic drugs (AEDs: *t*[32] = 1.00, *P* = 0.32). All patients were in the chronic stage postsurgery (at least 1 year postsurgery), although the right TLE patients were slightly more chronic than the left TLE group (*t*[32] = 2.11, *P* = 0.04). All patients had late-onset epilepsy, no history of other neurological or psychiatric disorders, were left language dominant based on the results of the WADA test and had epilepsy arising from unilateral mesial temporal sclerosis. All were native English speakers, right handed with normal or corrected-to-normal vision. The study was approved by the local ethics board.

**Table 1:** Demographic information and background behavioral testing for the control participants and TLE patient groups. Values are expressed as mean (standard deviation)

	Controls *n* = 20	Left TLE *n* = 17	Right TLE *n* = 17
Age (years)	38.2 (12.2)	42.9 (11.6)	44.9 (9.2)
Education (years)	17.1 (2.2)	14.8 (2.5)	15.6 (3.4)
Gender (M:F)	11:9	9:8	9:8
Age at surgery (years)	–	38.3 (11.2)	37.3 (10.1)
Years since surgery	–	4.6 (4.1)	7.5 (3.9)
Age at diagnosis (years)	–	15.7 (8.0)	15.6 (8.6)
Epilepsy duration (years)	–	22.6 (11.2)	21.6 (9.9)
Number of AEDs	–	2.5 (1.2)	1.8 (1.3)
Volume resected (mm^3^)	–	39.3 (8.7)	64.9 (22.3)
Background behavioral testing			
WASI			
Overall	–	91.9 (12.1)	101.4 (14.9)
Verbal	–	84.0 (18.2)	98.2 (8.4)
Matrix Reasoning	–	100.3 (13.7)	104.1 (11.4)
Rey complex figure			
Copy (max = 36)	35 (3)	35 (1)	35 (1)
Immediate (max = 36)	21 (8)	17 (7)*	14 (8)*
Delayed (max = 36)	22 (9)	16 (7)*	14 (8)*
Digit span			
Forward (max = 9)	6.8 (0.9)	6.1 (1.1)	6.8 (1.1)
Backward (max = 9)	4.6 (1.1)	4.4 (0.9)	4.2 (1.1)
Camden episodic memory			
Words (max = 25)	23 (2)	20 (3)*	22 (3)
Faces (max = 25)	23 (1)	21 (5)	21 (3)
Picture naming (max = 38)	34 (6)	26 (6)*^,^**	30 (4)*
WPM (max = 46)	43 (2)	39 (4)*	41 (3)
Synonym judgment (max = 96)	89 (6)	79 (6)*^,^**	87(6)

*P < 0.05 versus controls; **P < 0.05 versus TLE group.

### Control Participants

The pattern of brain activation and behavioral performance in the scanner of the left and right TLE patients were compared separately to a group of 20 age-matched control participants (controls versus left TLE: *t*[35] = 1.19, *P* = 0.24; controls versus right TLE: *t*[35] = 1.85, *P* = 0.07). The TLE patients had completed marginally less formal education than controls, consistent with their long-standing neurological condition (controls versus left TLE: *t*[35] = 3.01, *P* = 0.005; controls vs. right TLE: *t*[35] = 1.69, *P* = 0.10). All control participants were right-handed, native English speakers and who had normal or corrected-to-normal vision. Control participants also underwent extensive neuropsychological testing to screen for any undiagnosed cognitive abnormalities. The experiment was approved by the local ethics board.

### Stimuli and Task

**Figure 1.**
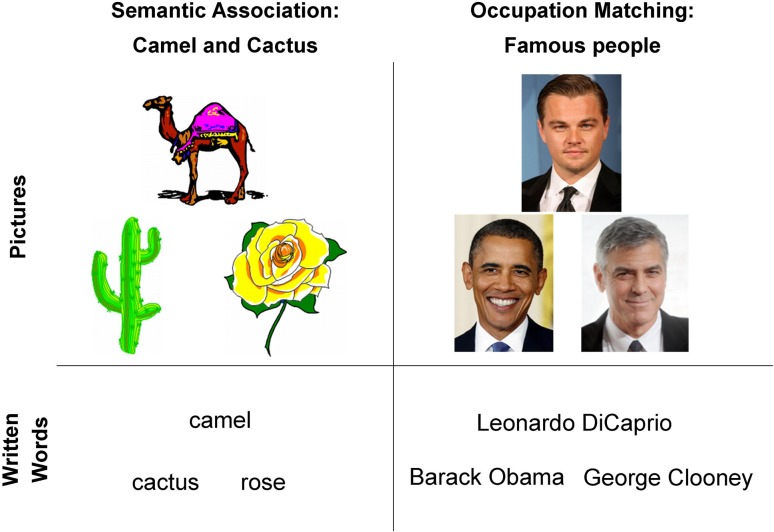
Examples of stimuli from the 4 semantic conditions. Stimuli were presented as pictures or written words.

Participants underwent 2 tasks in the scanner: a semantic association test and a test of occupation matching in famous people (Fig. 1). The semantic association task was the Camel and Cactus test (Bozeat et al. 2000; Visser et al. 2012). On each trial participants were presented with a probe item and asked to decide which of 2 alternatives was semantically related. The second task was an occupation judgment task. On each trial participants were presented with a probe item and asked to decide which of 2 alternatives had the same occupation. For each trial on the occupation matching task, all items shared the same gender. To investigate potential laterality differences due to modality, both the semantic association task and the occupation matching task were presented as written words and pictures. The written word condition was included to reflect a “left lateralized” semantic task based on the previous literature (Gainotti 2012; Rice, Hoffman, and Lambon Ralph 2015; Rice, Lambon Ralph et al. 2015), pictures of famous faces were included to reflect a “right lateralized” semantic task, again based on the predictions from the previous literature (Gainotti 2007; 2012). However, we failed to see any differences between pictures of objects and famous faces (see Supplementary Figs S1 and S2), therefore these 2 conditions were collapsed to form a single “picture” condition. Different items were used in the word and picture versions to avoid priming effects. Each condition contained 33 items.

In addition to the semantic conditions, a visual matching task was used to account for low level visual effects and provide an attention-demanding baseline condition. Baseline items were generated by visually scrambling items from each of the 4 semantic conditions; these were created using the Java Runtime Environment (www.SunMicrosystems.com) by scrambling each image into 120 pieces and rearranging them in a random order. For the written word trials (CCw, famous names) the baseline condition was scrambled words and for the picture trials (CCp, famous faces) the baseline condition was scrambled versions of the picture stimuli. The baseline task was presented in the same way as the experimental condition, with 1 probe item and 2 choices. One of the choices was identical to the target item and the other was an inverted version of the target. Participants were asked to decide which of the 2 choices was identical to the probe item.

### Procedure

TLE patients and controls underwent 4 functional scans, each with a total scan time of 8.45 min. During scanning, stimuli were presented in a block design using E-Prime software (Psychology Software Tools). Each functional scan contained stimuli from one semantic condition (CCw, CCp, famous names or famous faces) and from the relevant baseline condition (scrambled pictures or scrambled words). This was done to avoid task switching effects in the scanner. Each block contained 3 trials from 1 experimental condition. Each stimulus and the response screen were presented for 5000 ms, with an interstimulus interval of 500 ms. The 2 experimental conditions (semantic and baseline) were sampled 11 times per functional scan in a counterbalanced order, giving a total of 22 blocks per scan. The order of the scans was randomized and counterbalanced across participants. Stimuli were presented visually via a mirror mounted on the head coil, angled at a screen at the foot of the scanner bed. All participants underwent practice trials before beginning the scan to familiarize them with the tasks.

To test whether any upregulation in activation in the TLE patients were in regions that respond to increased task demands, we manipulated task difficulty for the control participants. In addition to the 4 functional scans in which stimuli were presented at the same rate as for the patients (Controls [Matched]), control participants also completed an additional 4 functional scans at a faster presentation speed (Controls [Speeded]). The same number of trials was presented as described above, but difficulty was increased by presenting stimuli twice as quickly, at 2500 ms intervals. Different stimuli were used in the slower and faster versions of the task by switching modality of presentation. For example, trials which were presented as pictures in the slow scan were presented as words in the faster scan. The slower and faster functional scans in the control participants were interleaved to avoid any habituation to the speed of presentation. Most control participants reported that the faster speed was more challenging but was not so difficult as to prevent them from completing the task (see also Behavioral Results).

Due to a technical issue with the button box in the scanner, accuracy data could only be recorded in 16 out of 17 left TLE patients and 19 out of 20 control participants. Similarly, reaction time data could only be recorded in 15 out of 17 left TLE patients and 19 out of 20 control participants (see Behavioral Results for full details).

### Imaging Parameters

Traditionally, imaging the ventral ATLs has been problematic because of a number of technical issues including the nature of the baseline contrast tasks as well as gradient-echo EPI signal drop-out and distortion (Devlin et al. 2000; Visser, Jefferies et al. 2010). These issues have been tackled through recent methodological developments (Embleton et al. 2010; Visser, Embleton et al. 2010). In the current study, the core semantic task was contrasted against an active baseline using dual-echo EPI imaging to improve signal in the vATLs (Halai et al. 2014).

All scans were acquired on a 3 T Phillips Achieva scanner, with a 32-channel head coil with a SENSE factor of 2.5. A dual-echo EPI sequence was used to improve signal-to-noise (SNR) in the vATLs (Halai et al. 2014). Using this technique, each scan consisted of 2 images acquired simultaneously with different echo times: a short echo optimized to obtain signal from the vATLs and a long echo optimized for good whole-brain coverage. The sequence included 31 slices covering the whole brain with repetition time (TR) = 2.8 s, echo times (TE) = 12 and 35 ms, flip angle = 85°, FOV = 240 × 240 mm^2^, resolution matrix = 80 × 80, slice thickness = 4 mm, voxel size = 3 × 3 × 4 mm^3^. All functional scans were acquired using a tilt, up to 45° off the AC-PC line, to reduce ghosting artefacts in the temporal lobes. For the TLE patients, functional scans were collected in four 8.45 min runs; each run acquired 177 dynamic scans (including 2 dummy scans, which were excluded). For the control participants, an additional four 4.3 min runs were included, each acquiring 88 dynamic scans (including 2 dummy scans, which were excluded). To address field-inhomogenities, a B0 field-map was acquired using identical parameters to the functional scans except for the following: TR = 599 ms, TEs = 5.19 and 6.65 ms. A high resolution T1 weighted structural scan was acquired for spatial normalization, including 260 slices covering the whole brain with TR = 8.4 ms, TE = 3.9 ms, flip angle = 8°, FOV = 240 × 191 mm, resolution matrix = 256 × 206, voxel size = 0.9 × 1.7 × 0.9 mm^3^.

### fMRI Analysis

Analysis was carried out using SPM8 (Wellcome Department of Imaging Neuroscience, London; www.fil.ion.ucl.ac.uk/spm).

### Automated Lesion Identification Procedure

**Figure 2.**
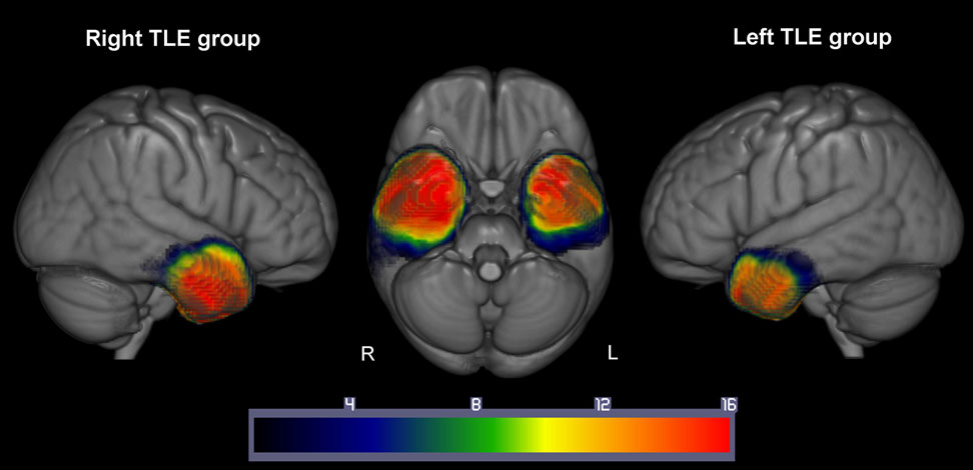
Resection overlap map for the 17 left and 17 right TLE patients. Overlap of the resection areas defined by the Seghier et al. (Seghier et al. 2008) method. Left TLE patients overlap is shown on the right of the image, right TLE patients overlap is shown on the left of the image. Color bars indicate the number of patients with resection in that area. Warmer colors = greater overlap, cooler colors = less overlap.

Automated outlines of the resection area were generated using Seghier et al. (2008) modified segmentation-normalization procedure, which is designed for use with brain-injured patients and which identifies areas of lesioned tissue. Data from both the TLE patients and the control participants were subjected to the automated lesion identification procedure. Segmented images were smoothed with an 8 mm full-width half maximum Gaussian kernel as recommended by Seghier et al. (2008) and submitted to the automated lesion identification and definition modules using the default parameters. The automated method involves initial segmentation and normalizing into tissue classes of grey matter, white matter, CSF and an extra tissue class which codes for the presence of the resection area. After smoothing, voxels that emerge as outliers relative to normal participants are identified and the union of these outliers provides the “fuzzy lesion map,” from which the resection outline is derived. The generated images were used to create the resection overlap map in Figure 2. For our patient sample, in order to ensure that the algorithm correctly identified the resection area as an extra class of tissue (rather than as CSF); the procedure was run twice for the TLE patients. The first iteration was run using the default settings in the toolbox; on the second iteration the default mask was changed to correspond to the output from the first iteration. This constrained the algorithm onto the resection area and allowed a more precise segmentation of the resection area. Overall, the right TLE patients resection volume was larger than that in the left TLE patients (Table 1; *t*[32] = 4.42, *P* < 0.0001). This is in keeping with the current surgical standards whereby resections to the left hemisphere are more conservative to avoid disruption to the language centers (Wiebe et al. 2001).

### Preprocessing

The functional images from the short and long echoes were averaged using custom MATLAB code (Halai et al. 2014). These combined images were realigned (for individual subject motion correction) and unwarped (for field-map correction). The mean functional image was then coregistered to the individual participant’s T1 structural scan. The data were then spatially normalized using the deformation fields generated in the Seghier method (see above) to MNI space and smoothed using an 8 mm Gaussian FWHM kernel. The normalized data were then masked to only include grey matter voxels (excluding those in the white matter and cerebral–spinal fluid); this grey matter mask was included as an explicit mask during the first level analysis. By default, SPM also employs an implicit mask which removes voxels which respond in the lowest 20% with respect to the mean signal across the brain. This step removes regions with low signal due to magnetic susceptibility and signal distortion. However, given our a priori hypotheses about the ATLs, a region with high magnetic susceptibility, and the fact that dual-echo acquisition was employed to reduce signal loss, this default threshold was removed so that all voxels would be included in the analysis (Halai et al. 2014).

At the individual subject level, contrasts of interest were modeled using a box-car function convolved with the canonical hemodynamic response function. Low frequency drifts were removed using a high-pass filter of 128 s. In each run, 2 separate regressors were modeled: 1) semantic condition (either: CCp, CCw, Famous Faces, Famous Names) and 2) baseline condition (either scrambled pictures or scrambled words).

### Whole Brain Analysis

The semantic and baseline regressors described above were used to create separate contrasts for the picture trials (pictures > scrambled pictures) by averaging across the CCp and famous face functional runs. The same method was followed to create a contrast for the written word trials (written word > scrambled words) by averaging across the CCw and famous name functional runs. For the whole brain analysis, individual contrast maps were entered into a second-level random effects analysis using one sample *t*-tests. This whole-brain map was thresholded at *P* < 0.001 at the voxel level, with a FWE-corrected cluster threshold of *P* < 0.05.

### ROI Analyses

Given the specific hypothesis regarding upregulation in the contralateral hemisphere after unilateral ATL resection, an a priori ROI analysis was employed to assess activation in the left and right vATLs. Peak coordinates were taken from a separate study on semantic processing that employed a written word task (Binney et al. 2010) (MNI: −15 −30; 36 −15 −30). A right vATL peak was created using the homologous coordinates. The comparisons of interest were between activation in the left vATL in the right TLE group versus Controls (Matched) and in the right vATL in the left TLE group versus Controls (Matched). Activations for the manipulation of difficulty in control participants (Controls [Matched], Controls [Speeded]) were also compared in the left and right vATLs separately. To explore the possibility of upregulation in cognitive (semantic) control regions after unilateral ATL resection, 2 additional ROIs were created using peaks from previous studies of semantic and cognitive control. The first peak was centered in the pars triangularis portion of the IFG (BA45) and was taken from a meta-analysis of semantic control. This region responded more to hard > easy semantic trials (MNI: −45 19 18; 47 23 26) (Noonan et al. 2013). The second peak was centered on the pars orbitalis portion of the IFG (BA47) and was taken from a study of semantic control; this region has been associated with controlled retrieval of semantic knowledge (MNI: −45 27 −15; 45 27 −15) (Badre et al. 2005).

## Results

### Behavioral Results

In the scanner, participants made semantic association judgments for the 4 semantic conditions and similarity judgments for the perceptual baseline conditions (Table 2). In the written word domain, comparisons between the left TLE patients and the Controls (Matched) condition revealed that the left TLE patients were significantly less accurate on the written word semantic task compared with controls (*t*[33] = 3.43, *P* = 0.002). There were no group differences on the nonsemantic baseline task (*t*[33] = 1.01, *P* = 0.32). The left TLE subgroup were slower compared with controls on both tasks (semantic: *t*[32] = 5.77, *P* < 0.0001; control: *t*[32] = 2.58, *P* = 0.02). Comparisons between the right TLE patients and the Controls (Matched) group for written words showed no group differences in terms of accuracy (semantic: *t*[34] = 1.85, *P* = 0.07; baseline: *t*[34] = 0.75, *P* = 0.46). As with the left TLE subgroup, the right TLE subgroup were slower compared with controls across both tasks (semantic: *t*[34] = 6.36, *P* < 0.0001; baseline: *t*[34] = 5.80, *P* < 0.0001). Comparisons within the left and right TLE subgroups revealed no significant differences in terms of accuracy (semantic: *t*[31] = 1.66, *P* = 0.11; baseline: *t*[31] = 1.70, *P* = 0.10) or reaction time (semantic: *t*[30] = 0.07, *P* = 0.95; baseline: *t*[30] = 1.82, *P* = 0.08) indicating that both groups were similarly impaired for the written word trials.

**Table 2:** In-scanner behavioral results. Mean accuracy (%) and correct response times (ms) across the experimental conditions (standard deviation in parenthesis). Note for the TLE patients and Controls (Matched) condition, stimuli were displayed on screen for 5000 ms, for the Controls (Speeded) condition stimuli were displayed for 2500 ms.

	Written word	Picture	Scrambled written	Scrambled picture
% Correct				
Left TLE	88 (7)*	78 (7)	91 (4)	93 (7)
Right TLE	91 (6)	79 (6)	88 (7)	92 (7)
Controls (Matched)	94 (5)	81 (6)	89 (6)	91 (10)
Controls (Speeded)	83 (11)**	75 (9)**	80 (9)**	81 (13)**
Correct response time (ms)				
Left TLE	2387 (378)*	2427 (344)*	2111 (422)*	2098 (468)*
Right TLE	2396 (340)*	2520 (323)*	2329 (269)*	2307 (313)*
Controls (Matched)	1714 (303)	1811 (262)	1791 (286)	1833 (317)
Controls (Speeded)	1367 (150)	1272 (112)	1336 (130)	1346 (130)

*Significant difference between the TLE group vs. Control (Matched)—independent *t*-test. **Significant difference between the 2 control conditions—paired *t*-test.

In the picture trials, comparisons between the left TLE patients and Controls (Matched) revealed no differences in accuracy between the 2 groups for the semantic task (*t*[33] = 1.58, *P* = 0.12) or the baseline task (*t*[33] = 0.72, *P* = 0.48). However, the left TLE subgroup were slower compared with controls for the semantic task (*t*[32] = 5.93, *P* < 0.0001) and showed a trend towards being slower for the baseline task (*t*[32] = 1.97, *P* = 0.06). Comparisons between the right TLE patients and the Controls (Matched) showed the same pattern: there were no differences between the groups in terms of accuracy for the semantic task (*t*[34] = 1.18, *P* = 0.25) or the baseline task (*t*[34] = 0.25, *P* = 0.80), but there was a nonspecific slowing on both tasks (semantic: *t*[34] = 7.27, *P* < 0.0001; baseline: *t*[34] = 4.51, *P* < 0.0001). As with the written word tasks, comparisons within the left and right TLE subgroups for the picture trials revealed no significant differences in terms of accuracy (semantic: *t*[31] = 0.47, *P* = 0.64; baseline: *t*[31] = 0.58, *P* = 0.57) or reaction time (semantic: *t*[30] = 0.79, *P* = 0.44; baseline: *t*[30] = 1.50, *P* = 0.14).

Importantly, the lack of differences in the performance of the left versus right TLE group could not be accounted for by the time since surgery. Overall, the right TLE group had a longer period of recovery since surgery compared with the left TLE group (right TLE group: 7.5 years vs. left TLE group: 4.6 years; Table 1). To explore the potential impact of number of years since surgery on semantic performance, we ran ANCOVAs on the patient data including this variable as a covariate. There was no change in the direction of the results reported above for the in-scanner behavioral performance. Both left and right TLE groups performed the picture and written word semantic tasks with equal behavioral performance, both in terms of accuracy and correct response time. There was no effect of the covariate on performance. Full results for this analysis can be found in Supplementary Table S1.

Comparisons between the 2 presentation speeds confirmed that, as intended, speeding up control participants increased the difficulty of the semantic and baseline tasks, reflected in reduced accuracy in both the picture (semantic: *t*[18] = 3.92, *P* = 0.001; baseline: *t*[18] = 4.99, *P* < 0.0001) and written word condition (semantic: *t*[18] = 4.38, *P* < 0.0001; baseline: *t*[18] = 5.47, *P* < 0.0001).

### Whole Brain Analyses

#### Written Word Tasks

**Figure 3.**
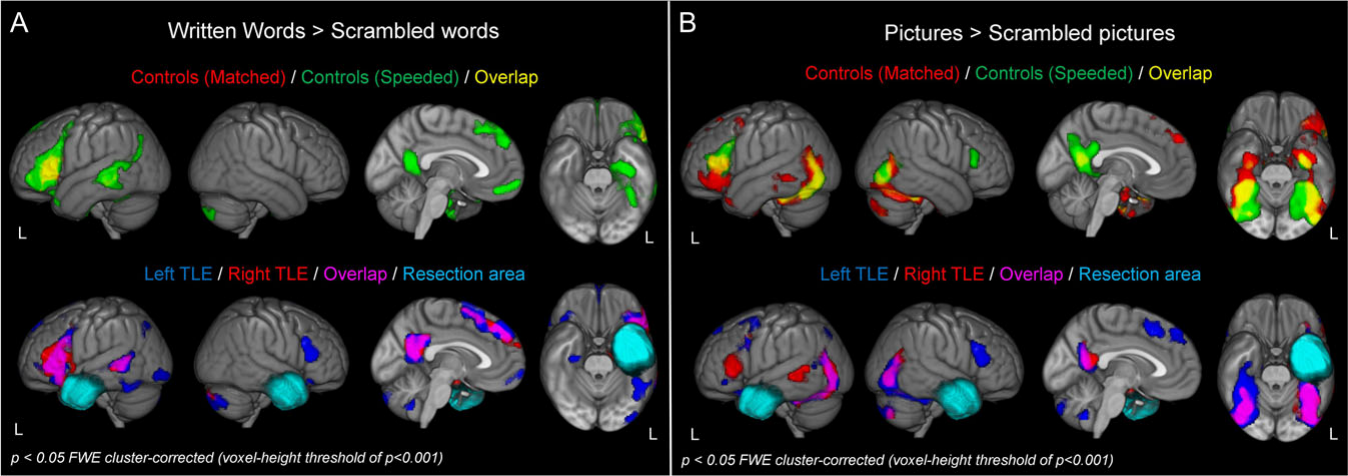
Whole brain results for the 2 semantic tasks. (A) Whole brain maps for the written word > scrambled word contrast for the 2 control conditions (Top: Matched—red, Speeded—green) and the 2 TLE patient groups (Bottom: left TLE—blue, right TLE—red). Resection areas for the 2 patients groups are shown in cyan; the resection area for the right TLE group is not displayed on the ventral view to illustrate the right vATL activation for the left TLE group. (B) Whole brain maps for the picture > scrambled picture contrast.

Regions involved in the written word tasks in the control participants and patients were identified using the whole brain contrasts “Written Words (CCw + Names) > Scrambled Words.” Peak activations for the whole brain contrasts are listed in Supplementary Table S2. Figure 3a shows an exclusively left lateralized network activated by control participants by the written word semantic task. For control participants at the matched speed (top, red) this included the left IFG and posterior middle temporal gyrus. At the faster speed, controls showed an increase in the extent of activation across the left hemisphere (top, green), particularly in the left IFG, posterior middle temporal gyrus and fusiform gyrus extending into the left vATL. Medial structures including the dorsomedial prefrontal cortex, orbitofrontal cortex and precuneus were also implicated. For the left TLE patients, written word semantic tasks activated broadly the same areas as control participants (although not extending into the left vATL because of the nature of the resection). In addition to these left hemisphere structures, left TLE patients also showed activation to written words in the right IFG (pars triangularis and pars orbitalis). For right TLE patients, written word tasks activated a largely left-lateralized network, which was very similar to the pattern of response seen in control participants.

#### Picture Tasks

Regions involved in the picture tasks in control participants and patients were identified using the whole brain contrast “Picture (CCp + Faces) > Scrambled Pictures.” Peak activations for the whole brain contrasts are listed in Supplementary Table S3. Figure 3b shows a widespread bilateral network of regions activated. For control participants at the matched speed (top, red) this included the length of the temporal lobes bilaterally, extending the length of the fusiform and inferior temporal gyri into the vATLs. At the slower speed, activation in the left IFG, bilateral posterior middle temporal gyrus and precuneus were also found. At the faster speed (green), control participants showed a greater extent of activation in all these areas, particularly in the precuneus and left IFG. Additional activation was also observed in the right IFG. For the left TLE patients, visual semantic tasks activated similar areas to control participants, with the notable exception of left IFG; the right IFG was also active, however. For the right TLE patients, again similar activation to the control participants was shown, and for these patients activation was found in the left but not right IFG.

### ROI Analysis

Whole brain results illustrate that the overall pattern of activation between postsurgical TLE patients and controls are broadly similar. Next, we tested whether TLE patients were over-activating or under-activating particular areas of interest in comparison to controls. Over-activation of a region may indicate a compensatory mechanism whereas under-activation may indicate a negative impact.

**Figure 4.**
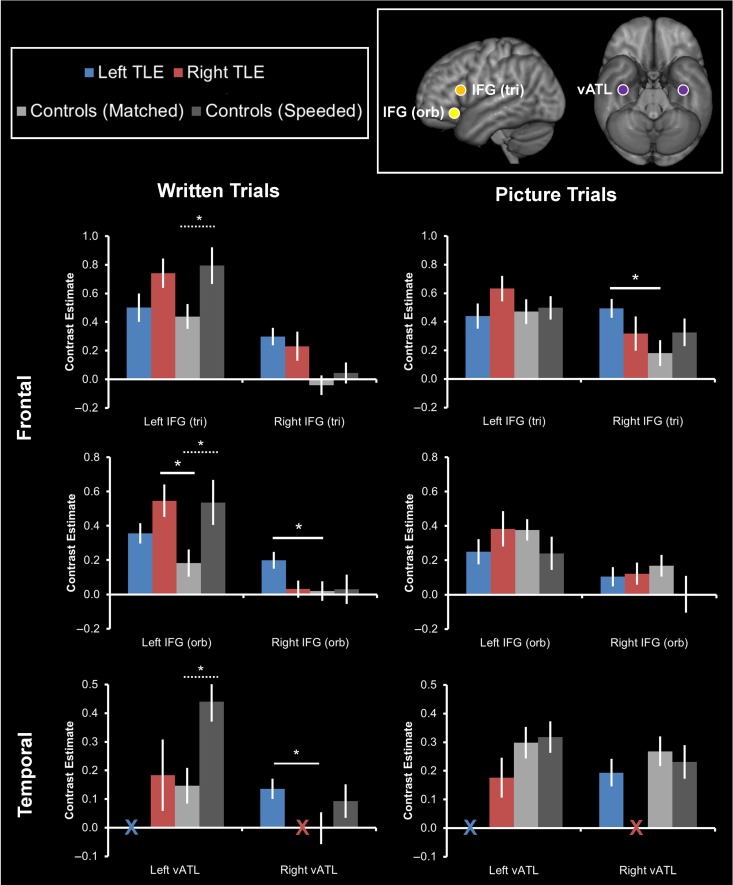
A priori ROI results for the picture and written word semantic tasks. Contrast estimates for each task are shown over the relevant baseline condition for the left and right TLE patients and the 2 control participant conditions (Matched vs. Speeded). Error bars denote standard error. vATL coordinates taken from Binney et al. (2010), IFG (triangularis) coordinates taken from Noonan et al. (2013), IFG (orbitalis) coordinates taken from Badre et al. (2005)—ROI positions are shown in the top right panel. “X” indicates where data are unavailable due to surgical resection. Full line = significant differences between the left or right TLE group and the Controls (Matched) condition, dashed line = significant differences between the 2 control conditions (Matched vs. Speeded).

Figure 4 shows the a priori ROI results for all participants. For the IFG ROI analysis, the analyses of interest were between-subjects ANOVAs comparing activation changes across the hemispheres (left, right) between the 2 TLE groups and Controls (Matched). For the ATL region, given the lack of data for the TLE subgroups in one hemisphere, the analysis of interest was independent *t* tests comparing activation changes between the 2 TLE subgroups and Controls (Matched) in the remaining hemisphere. Finally, paired *t*-tests comparing activation changes as a function of presentation speed in controls. By comparing the pattern of activation between the 2 speeds in controls, we are able to address the secondary question of this study, which was to explore whether patients use a separate set of regions in order to carry out semantic memory tasks after surgery or whether they upregulate activity in the same regions engaged by healthy individuals during more challenging semantic processing.

#### Written Word Tasks

During written word processing, in all 3 left hemisphere ROIs (Fig. 4) there was a significant upregulation of activation in the Controls (Speeded) condition compared with the Controls (Matched) condition (paired *t*-tests = vATL: *t*[19] = 4.27, *P* < 0.0001; pars triangularis: *t*[19] = 3.07, *P* = 0.006; pars orbitalis: *t*[19] = 2.62, *P* = 0.017), indicating that controls increased activity across the semantic network when task demands increased.

Compared with controls, TLE patients showed increased activation in a number of these areas. The right TLE group showed greater activation than controls in left IFG but not the left ATL. In direct contrast to this, the left TLE group showed significant upregulation of activation compared with Controls (Matched) only in the right hemisphere. Specifically, in the IFG (pars orbitalis) a 2-way Group*Hemisphere ANOVA showed a significant Group*Hemisphere interaction (*F*[2, 51] = 8.27, *P* = 0.001). This was driven by an upregulation of activation in the left IFG in the right TLE group compared with Controls (Matched; *t*[35] = 2.96, *P* = 0.006). In contrast to this, the left TLE group showed an upregulation in the right IFG compared with Controls (Matched; *t*[35] = 2.34, *P* = 0.025) and the right TLE group (*t*[35] = 2.38, *P* = 0.023). In the ATL ROI there was no corresponding upregulation of activation in the left ATL in the right TLE group compared with Controls (*t*[35] = 0.27, *P* = 0.79). There was a significant increase in activation in the right ATL in the left TLE group (*t*[35] = 2.04, *P* = 0.049).

### Picture Tasks

In contrast to the group differences shown in the written word condition, there were very few group differences in the picture condition (Fig. 4). Comparisons between the 2 speeds in controls revealed no changes in activation in any of the ROIs, in either the left or right hemisphere during picture trials. However, a 2-way Group*Hemisphere ANOVA performed on the IFG (pars triangularis) data showed a significant Group*Hemisphere interaction (*F*[2, 51] = 4.2, *P* = 0.02).This was driven by an upregulation of activation in the right IFG in the left TLE group compared with Controls (Matched; *t*[35] = 2.73, *P* = 0.01). The right TLE group showed no significant differences to the Controls (Matched) condition in any ROI.

## Discussion

This study explored neural reorganization of semantic function in patients with unilateral ATL resection for TLE. The semantic representation system is bilaterally organized, and is robust to unilateral damage/perturbation; however, the neural underpinnings of this mechanism have yet to be fully elucidated. One view holds that residual semantic performance is maintained via upregulation of activation in the contralateral ATL (Schapiro et al. 2013; Binney and Lambon Ralph 2015; Jung and Lambon Ralph 2016). An alternative, but not mutually exclusive, suggestion is that task performance is maintained via upregulation of cognitive/semantic control regions (Backes et al. 2005; Maccotta et al. 2007; Wong et al. 2009; Bonelli et al. 2012; Sidhu et al. 2013). Here we investigated the maintenance of semantic performance for both word and picture stimuli in a group of postsurgical left and right TLE patients, using an imaging protocol that improves signal in the vATLs (Visser, Embleton et al. 2010; Halai et al. 2014). The principal finding was of dynamic changes in the semantic network after unilateral resection—supporting both types of proposed resilience mechanisms. In particular, differential activation between patients and control participants were shown in the IFG and vATLs. These changes were not uniform; rather the degree of upregulation changed depending on the task being undertaken. Semantic tasks presented as pictures elicited bilateral activation in both the control participants and TLE patients. The distribution of activation was very similar, with the only group difference being stronger right IFG activation in the left TLE patients compared with controls. These minimal group differences in the fMRI data matched the pattern of results in the behavioral data, which showed no differences in accuracy across the TLE patients and controls. In contrast, during written word semantic tasks, activation was strongly lateralized to the left hemisphere in both controls and patients (a pattern that has been observed in large-scale meta-analyses of the semantic fMRI literature in healthy participants: Rice, Lambon Ralph et al. 2015). More striking group differences between the patients and controls were revealed in this condition. The right TLE group upregulated the left IFG compared with controls; whereas, the left TLE group upregulated the homologous right IFG and right vATL. This differential fMRI activation profile in the left TLE group reflected the behavioral findings, which showed less accurate responses in the left TLE group in written word tasks. The results suggest, therefore, that 1) the patients upregulate both the representational (contralateral ATL) and executive control (IFG) systems in a manner that seems to reflect intrinsic mechanisms which make the healthy system resilient to increased demand (cf. the speeded condition in healthy individuals), but 2) these effects interact with both task modality and the side of resection.

The semantic representation system is intrinsically robust to unilateral damage compared with bilateral damage (Kluver and Bucy 1937; 1938; Terzian and Ore 1955; Wilkins and Moscovitch 1978; Lambon Ralph et al. 2010, 2012). Unilateral resection results in a mild semantic impairment which is most reliably shown through slower response times on semantic tasks (Wilkins and Moscovitch 1978; Lambon Ralph et al. 2012; Rice et al. 2018). The adaptive properties of the semantic system after unilateral resection/perturbation have been ascribed to the upregulation of activation in the remaining contralateral ATL which helps to maintain the robustness of the system (Schapiro et al. 2013; Binney and Lambon Ralph 2015; Jung and Lambon Ralph 2016). This mechanism of contralateral upregulation has also been proposed as a mechanism within the medial temporal lobes for the maintenance of episodic memory function (Bonelli et al. 2012, 2013; Sidhu et al. 2013). Here, we showed some changes in the remaining ATL in TLE patients, although these changes interacted with the task demands. Semantic tasks presented as pictures elicited a strongly bilateral representation in the left and right vATLs in healthy participants, in keeping with previous observations of tasks requiring a response to pictures (Rice, Lambon Ralph et al. 2015). Under increased task demands, no upregulation was seen in the left or right vATLs in control participants. Similarly, after resection of either the left or right ATL, no upregulation of activation in the remaining ATL was found in for patients. This implies that information that is represented bilaterally is more robust to unilateral damage, such that the contralateral ATL may retain the conceptual representations or sufficient noise-free fidelity needed to complete the task without the need for upregulation (Schapiro et al. 2013).

In contrast, written word semantic tasks elicited a strongly left lateralized network in healthy participants, which again is in line with previous neuroimaging results (Rice, Lambon Ralph et al. 2015). Under increased task demands in control participants, there was strong upregulation in the left vATL, but not the right. The right TLE group also activated a strongly left lateralized network during written word processing. In contrast, the left TLE patients activated the right vATL more than control participants (directly mirroring the 2 previous combined rTMS-fMRI explorations in healthy participants, which also utilized written word materials) (Binney and Lambon Ralph 2015; Jung and Lambon Ralph 2016), indicating reorganization of verbal semantic processing in these patients to make use of the contralateral ATL. These patients were significantly impaired in completion of the verbal task, implying that tasks that are relatively lateralized to one hemisphere are more vulnerable to unilateral damage/resection of the dominant hemisphere (see also, Schapiro et al. 2013). Although representations stored in the contralateral ATL do appear to be recruited in these patients, in this case they were not sufficient to maintain normal performance.

In addition to an intact ATL representational store, successful semantic cognition requires executive control processes that serve to shape this information in a task appropriate manner (Jefferies and Lambon Ralph 2006; Jefferies 2013; Rogers et al. 2015). After degradation of the semantic store (through unilateral ATL resection or perturbation), upregulation in controlled retrieval mechanisms in the bilateral IFG may become necessary in order to maintain normal task performance (Koylu et al. 2006; Bonelli et al. 2012; Sidhu et al. 2013). Here, reliable activation changes were revealed in 2 separate areas of the IFG. One cluster was located in pars orbitalis (BA47) and the second was located in pars triangularis (BA45). As in the vATL, activation changes in the IFG interacted with task modality. In the picture-based semantic task there were few group differences. More striking group differences were shown in the written word task; specifically, the right TLE group showed an upregulation of activation in the left IFG (pars orbitalis) compared with control participants. In contrast, left TLE patients increased activation in the right IFG, homologous to regions upregulated by right TLE patients. The differential IFG activation profile in TLE patients versus control participants accords with previous findings in postsurgical TLE patients (Backes et al. 2005; Koylu et al. 2006; Bonelli et al. 2012) and suggests that upregulation of semantic control regions may be important for supporting semantic processing in patients with ATL resection for TLE. It may be worth noting that the specific upregulated PFC regions were the pars orbitalis. Previous studies of healthy semantic function (Badre et al. 2005) found that these regions were crucial when the target semantic information was inherently weak and presumably required a form of “amplification.” This is consistent with the possibility that the patients’ long-term ATL dysfunction and later resection weakens their semantic representations which can be boosted via interaction with these ventral PFC semantic control regions.

A related question is whether the areas showing dynamic changes are specific to the patients or whether such changes are part of the normal mechanisms that are used in the healthy system. In other words, recovery may involve reoptimization of existing resources whereby contralateral regions that were already somewhat involved in supporting the affected functions upregulate their contribution to compensate for the damage. Evidence for this type of upregulation in the contralateral hemisphere has been found in the language/semantic domain after stroke (Leff et al. 2002; Warren et al. 2009) and for long-term recovery of episodic memory function after resection for TLE (Bonelli et al. 2012, 2013; Sidhu et al. 2016). In neurologically intact participants, this same mechanism has been demonstrated for semantic aspects of language (Sharp et al. 2004). Brownsett et al. (2014) went one step further and showed that regions in the frontal lobes that were upregulated during language processing in poststroke aphasia overlapped with regions that healthy controls upregulated when listening to degraded speech. This supports the general view that patients increase engagement of demand-related prefrontal regions to compensate for damage elsewhere. Future research should explore the interaction between the frontal and temporal lobe semantic systems in maintaining normal semantic performance, not only in patient populations but in the healthy semantic system.

Finally, we note that from the current study it is not possible to determine whether the differences in activation between the TLE patients and control participants were caused by the surgery itself or whether activation patterns in TLE patients were affected prior to surgery as a result of long-standing epilepsy (or a combination of the 2). The purpose of the current study was to establish changes in the function of the semantic network in a group of chronic postsurgery TLE patients. The purpose of the study was not to explore the root causes behind any patient versus control group differences, for which presurgical data in the patients is required. Follow-up studies can use this paradigm to explore semantic abilities both before and after TLE resection with respect to both the patients’ semantic performance and its neural bases. These types of studies would allow us to explore whether there are semantic deficits presurgery, and whether or not these are exacerbated by the resection surgery. The effect of surgery on different semantic tasks (e.g., pictures vs. written words) should also be a future avenue for research.

## Supplementary Material

Supplementary Data
