# Supplementary material for: Revealing the Dynamic Modulations That Underpin a Resilient Neural Network for Semantic Cognition: An fMRI Investigation in Patients With Anterior Temporal Lobe Resection
Source: Cereb Cortex. 2018 Jun 6;28(8):3004–16. doi: 10.1093/cercor/bhy116 (PMC6041810; doi:10.1093/cercor/bhy116)
Supplement: Supplementary Data [file bhy116_supplementarymaterials.zip › bhy116_RiceCaswellMooreLambonRalphHoffman_SupplementaryMaterialsCC.docx]

**Rice, Caswell, Moore, Lambon Ralph & Hoffman.**

**Supplementary materials**

***Supplementary Figure 1: Whole brain results for the two picture semantic tasks.*** *(A) Whole brain maps for the CCp > scrambled picture contrast for the two control conditions (Top: Matched – red, Speeded- green) and the two TLE patient groups (Bottom: left TLE – blue, right TLE – red). Resection areas for the two patients groups are shown in cyan; the resection area for the right TLE group is not displayed on the ventral view to illustrate the right vATL activation for the left TLE group. (B) Whole brain maps for the famous faces > scrambled picture contrast. All maps are thresholded at p < 0.05 FWE cluster-corrected (voxel height threshold of p < 0.001).*


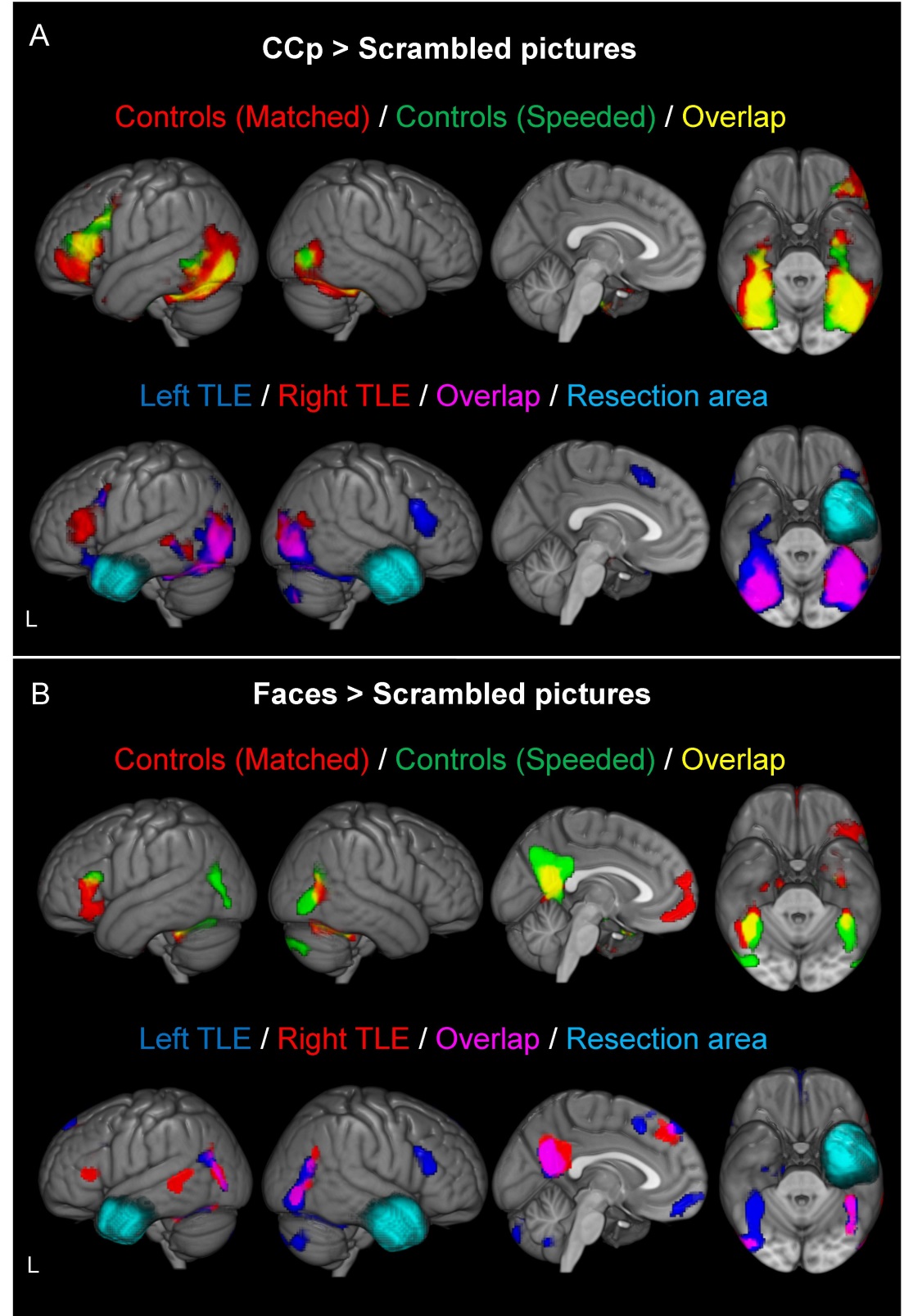


***Supplementary Figure 2: Whole brain results for the two written word semantic tasks.*** *(A) Whole brain maps for the CCw > scrambled word contrast for the two control conditions (Top: Matched – red, Speeded- green) and the two TLE patient groups (Bottom: left TLE – blue, right TLE – red). Resection areas for the two patients groups are shown in cyan. (B) Whole brain maps for the famous names > scrambled word contrast. The resection area for the right TLE group is not displayed on the ventral view to illustrate the right vATL activation for the left TLE group. All maps are thresholded at p < 0.05 FWE cluster-corrected (voxel height threshold of p < 0.001).*

*
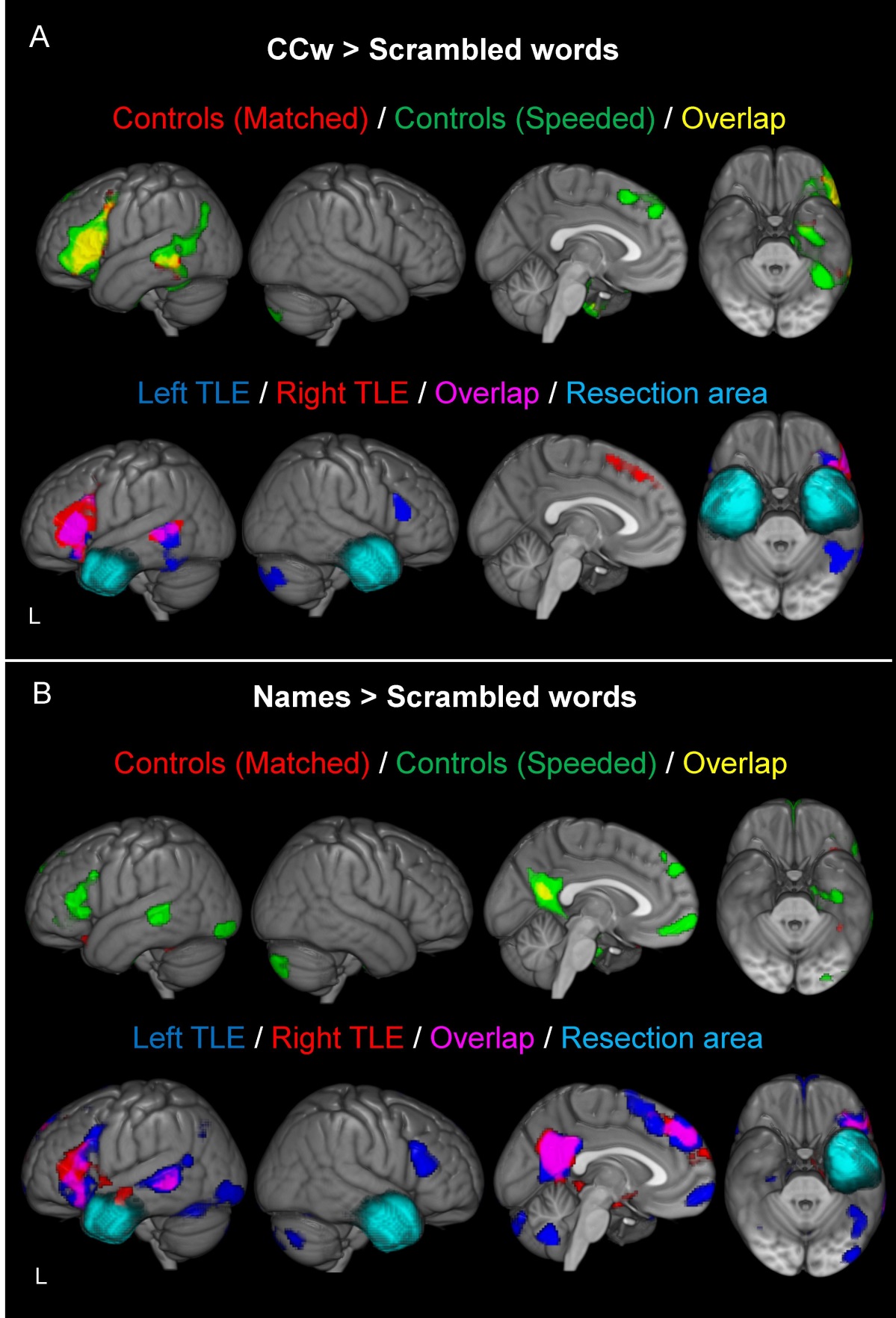
*

| **Supplementary Table 1: Comparison of statistical differences between the left and right TLE patients before and after controlling for the effect of number of years since surgery.** Data shown for accuracy (left) and correct reaction time (right) in the in-scanner behavioural semantic tasks. Written word task data reflect the data from the CCw and famous name conditions, picture task data reflect the data from the CCp and famous face conditions. The first column reiterates the t-test results between the two patient groups reported in the “Behavioural Results paragraph”. The second column reports the main effect of patient group from an ANOVA (left TLE vs. right TLE). The third column reports the significance of the main effect of patient group after controlling for time since surgery (years). The cells are colour coded according to the significance of the group main effect. Green indicates significance below p < 0.05, red indicates non-significance. | | | | | | |
| --- | --- | --- | --- | --- | --- | --- |
|  | **ACC** | | | **RT** | | |
|  | **Original t-test: left TLE, right TLE** | **ANOVA: left TLE, right TLE (n=33)** | **ANCOVA: left TLE, right TLE (n=33)** | **Original t-test: left TLE, right TLE** | **ANOVA: left TLE, right TLE (n=32)** | **ANCOVA: left TLE, right TLE (n=32)** |
| **Written word task** | t (31) = 1.66, p = 0.11 | F (1, 31) = 2.76, p = 0.11 | F (2, 30) = 1.19, p = 0.29  Covariate: F (1, 30) = 1.34, p = 0.26 | t (30) = 0.07, p = 0.95 | F (1, 30) = 0.005, p = 0.95 | F (2, 30) = 0.38, p = 0.85  Covariate: F (1, 30) = 0.10, p = 0.76 |
| **Picture task** | t (31) = 0.47, p = 0.64 | F (1, 31) = 0.22, p = 0.64 | F (2, 30) = 0.04, p = 0.84  Covariate: F (1, 30) = 0.34, p = 0.56 | t (30) = 0.79, p = 0.44 | F (1, 30) = 0.62, p = 0.44 | F (2, 30) = 0.44, p = 0.51  Covariate: F (1, 30) = 0.009, p = 0.93 |
|  | | | | | | |
|  | Significant main effect of group (left vs. right TLE) | | | | | |
|  | Main effect of group not significant | | | | | |

**Supplementary Table 2:** Peak coordinates for the whole brain written word > scrambled word contrast. All coordinates thresholded at p < 0.001 at the voxel level, p < 0.05 FWE-cluster corrected.

| **Written Words > Scrambled Words** | | | | | |
| --- | --- | --- | --- | --- | --- |
| **Region** | **MNI** | | |  |  |
|  | **X** | **Y** | **Z** | **Z value** | **Cluster size** |
| ***Left TLE*** | | | | | |
| Inferior Frontal Gyrus (Orbitalis) | 38 | 34 | -14 | 5.25 | 290 |
| Inferior Frontal Gyrus (Orbitalis) | -42 | 34 | -16 | 4.98 | 3411 |
| Mid Frontal Gyrus | -28 | 16 | 52 | 4.70 |  |
| Inferior Frontal Gyrus (Orbitalis) | -28 | 32 | -6 | 4.67 |  |
| Posterior Cingulum | 4 | -40 | 30 | 4.95 | 1196 |
| Precuneus | 0 | -56 | 28 | 4.57 |  |
|  | -6 | -58 | 14 | 4.56 |  |
| Inferior Frontal Gyrus (Triangularis) | 52 | 24 | 16 | 4.87 | 687 |
|  | 44 | 18 | 26 | 4.40 |  |
| Superior Medial Frontal Gyrus | 2 | 38 | 40 | 4.69 | 1501 |
|  | -2 | 46 | 42 | 4.55 |  |
| Supplementary Motor Area | -4 | 22 | 52 | 4.39 |  |
| Medial Occipital Gyrus | -34 | -68 | 40 | 4.68 | 462 |
| Angular Gyrus | -38 | -62 | 30 | 3.45 |  |
| Posterior Middle Temporal Gyrus | -58 | -46 | 2 | 4.67 | 1841 |
|  | -64 | -46 | -4 | 4.57 |  |
| Posterior Fusiform Gyrus | -48 | -60 | -20 | 4.15 |  |
| Cerebellum | 14 | -80 | -32 | 4.41 | 1750 |
|  | 42 | -70 | -38 | 4.40 |  |
|  | 16 | -86 | -42 | 4.16 |  |
|  | 6 | -56 | -42 | 4.25 | 305 |
| ***Right TLE*** | | | | | |
| Inferior Frontal Gyrus (Triangularis) | -46 | 24 | 20 | 5.41 | 4950 |
|  | -50 | 24 | 12 | 5.09 |  |
| Inferior Frontal Gyrus (Orbitalis) | -40 | 32 | -4 | 5.02 |  |
| Hippocampus | -30 | -18 | -16 | 5.10 | 874 |
|  | -16 | -10 | -18 | 4.26 |  |
| Putamen | -24 | 2 | -8 | 3.91 |  |
| Posterior Middle Temporal Gyrus | -62 | -42 | -4 | 4.44 | 708 |
|  | -60 | -48 | 4 | 4.14 |  |
|  | -52 | -56 | 10 | 3.35 |  |
| Precuneus | 4 | -60 | 24 | 4.38 | 1116 |
|  | 2 | -44 | 34 | 4.21 |  |
|  | -4 | -54 | 18 | 4.11 |  |
| Cerebellum | 22 | -86 | -34 | 4.14 | 892 |
|  | 14 | -80 | -30 | 3.98 |  |
|  | 28 | -72 | -42 | 3.89 |  |
| ***Controls (Matched)*** | | | | | |
| Inferior Frontal Gyrus (Triangularis) | -52 | 30 | 0 | 4.79 | 1367 |
|  | -48 | 24 | 12 | 4.45 |  |
| Inferior Frontal Gyrus (Opercularis) | -42 | 8 | 24 | 3.67 |  |
| Posterior Middle Temporal Gyrus | -52 | -38 | 6 | 3.95 | 374 |
|  | -58 | -44 | 2 | 3.62 |  |
|  | -60 | -34 | -14 | 3.50 |  |
| ***Controls (Speeded)*** | | | | | |
| Parahippocampal Gyrus | -22 | -22 | -18 | 5.59 | 2295 |
| Anterior Fusiform Gyrus | -34 | -12 | -40 | 4.80 |  |
|  | -44 | -22 | -36 | 4.62 |  |
| Inferior Frontal Gyrus (Triangularis) | -54 | 24 | 16 | 5.24 | 3976 |
| Inferior Frontal Gyrus (Orbitalis) | -40 | 44 | -6 | 4.90 |  |
|  | -50 | 36 | -16 | 4.84 |  |
| Precuneus | -4 | -54 | 10 | 5.20 | 491 |
| Cerebellum | 18 | -88 | -42 | 4.96 | 1008 |
|  | 12 | -80 | -32 | 4.25 |  |
|  | 34 | -72 | -38 | 4.07 |  |
| Posterior Middle Temporal Gyrus | -70 | -36 | -2 | 4.86 | 2002 |
|  | -52 | -48 | 14 | 4.52 |  |
|  | -50 | -64 | 20 | 4.13 |  |
| Gyrus Rectus | 0 | 44 | -16 | 4.72 | 413 |
|  | 0 | 62 | -10 | 4.09 |  |
| Superior Medial Frontal Gyrus | -6 | 34 | 50 | 4.26 | 889 |
|  | -8 | 44 | 42 | 4.02 |  |
|  | -4 | 50 | 36 | 3.92 |  |

**Supplementary Table 3:** Peak coordinates for the whole brain picture > scrambled picture contrast. All coordinates thresholded at p < 0.001 at the voxel level, p < 0.05 FWE-cluster corrected.

| **Pictures > Scrambled Picture** | | | | | |
| --- | --- | --- | --- | --- | --- |
| **Region** | **MNI** | | |  |  |
|  | **X** | **Y** | **Z** | **Z value** | **Cluster size** |
| ***Left TLE*** | | | | | |
| Posterior Fusiform Gyrus | 42 | -54 | -24 | 6.18 | 7132 |
| Inferior Occipital Gyrus | 46 | -76 | -10 | 5.90 |  |
| Cerebellum | 40 | -68 | -34 | 5.27 |  |
| Inferior Frontal Gyrus (Triangularis) | 50 | 20 | 20 | 6.04 | 1219 |
| Inferior Frontal Gyrus (Opercularis) | 42 | 12 | 38 | 4.00 |  |
| Inferior Frontal Gyrus (Triangularis) | 38 | 32 | 8 | 3.58 |  |
| Inferior Occipital Gyrus | -46 | -82 | -4 | 5.31 | 3279 |
| Posterior Fusiform Gyrus | -44 | -64 | -20 | 5.25 |  |
|  | -38 | -46 | -20 | 5.08 |  |
| Supplementary Motor Area | -4 | 16 | 48 | 4.83 | 1035 |
| Superior Frontal Gyrus | -12 | 44 | 48 | 4.06 |  |
| Superior Medial Frontal Gyrus | -2 | 46 | 48 | 3.98 |  |
| Cerebellum | -12 | -78 | -32 | 4.70 | 314 |
| Lingual Gyrus | -10 | -86 | -40 | 4.51 |  |
| Inferior Frontal Gyrus (Orbitalis) | -32 | 28 | -6 | 4.70 | 2287 |
| Insula | -42 | 16 | 28 | 4.63 |  |
| Middle Frontal Gyrus | -48 | 12 | 38 | 4.36 |  |
| Lingual Gyrus | -4 | -60 | 10 | 4.37 | 503 |
| Precuneus | 0 | -62 | 26 | 4.36 |  |
|  | -6 | -46 | 8 | 3.34 |  |
| Cerebellum | -4 | -58 | -48 | 4.31 | 279 |
|  | 8 | -54 | -48 | 4.12 |  |
| ***Right TLE*** | | | | | |
| Inferior Frontal Gyrus (Triangularis) | -46 | 24 | 8 | 5.30 | 2127 |
|  | -42 | 24 | 20 | 4.97 |  |
|  | -38 | 28 | 10 | 4.67 |  |
| Inferior Occipital Cortex | -40 | -70 | -14 | 5.12 | 4852 |
| Hippocampus | -18 | -8 | -16 | 4.81 |  |
|  | -28 | -6 | -16 | 4.79 |  |
| Inferior Occipital Lobe | 46 | -76 | -4 | 4.75 | 2092 |
| Posterior Fusiform Gyrus | 42 | -66 | -16 | 4.29 |  |
|  | 36 | -60 | -14 | 4.19 |  |
| Precuneus | -2 | -54 | 14 | 4.45 | 782 |
|  | -12 | -52 | 10 | 3.80 |  |
|  | 4 | -60 | 34 | 3.58 |  |
| Cerebellum | 14 | -78 | -34 | 4.26 | 746 |
|  | 22 | -78 | -36 | 4.13 |  |
|  | 40 | -76 | -50 | 4.10 |  |
| ***Controls (Matched)*** | | | | | |
| Anterior Inferior Temporal Gyrus | -36 | -6 | -42 | 5.36 | 5773 |
| Temporal Pole | -28 | 12 | -32 | 5.02 |  |
| Posterior Middle Temporal Gyrus | -44 | -62 | 16 | 4.82 |  |
| Middle Fusiform Gyrus | 36 | -38 | -26 | 5.18 | 4835 |
| Posterior Middle Temporal Gyrus | 56 | -58 | 2 | 4.84 |  |
| Posterior Inferior Temporal Gyrus | 50 | -56 | -26 | 4.75 |  |
| Inferior Frontal Gyrus (Triangularis) | -52 | 26 | 8 | 4.97 | 3123 |
| Inferior Frontal Gyrus (Orbitalis) | -44 | 34 | -14 | 4.84 |  |
|  | -54 | 32 | -4 | 4.72 |  |
| Superior Medial Frontal Gyrus | 0 | 54 | 36 | 4.02 | 403 |
| Superior Frontal Gyrus | -16 | 40 | 46 | 3.65 |  |
|  | -12 | 58 | 28 | 3.36 |  |
| Cerebellum | 36 | -74 | -50 | 3.90 | 320 |
|  | 34 | -84 | -34 | 3.66 |  |
| Precuneus | 2 | -56 | 16 | 3.82 | 280 |
| ***Controls (Speeded)*** | | | | | |
| Inferior Frontal Gyrus (Triangularis) | -44 | 28 | 6 | 5.69 | 2609 |
|  | -40 | 14 | 28 | 5.63 |  |
| Middle Frontal Gyrus | -28 | 12 | 42 | 4.31 |  |
| Posterior Fusiform Gyrus | -40 | -54 | -20 | 5.66 | 4816 |
|  | -42 | -48 | -30 | 5.63 |  |
|  | -36 | -62 | -14 | 5.27 |  |
| Posterior Fusiform Gyrus | 34 | -50 | -8 | 5.63 | 7418 |
| Precuneus | -6 | -54 | 10 | 5.50 |  |
| Cerebellum | 38 | -48 | -26 | 5.49 |  |
| Inferior Frontal Gyrus (Triangularis) | 52 | 28 | 10 | 5.41 | 471 |
| Anterior Fusiform Gyrus | -32 | -14 | -34 | 5.25 | 1245 |
| Hippocampus | -30 | -18 | -12 | 5.24 |  |
| Anterior Fusiform Gyrus | -30 | -8 | -40 | 4.59 |  |
